# Core-Genome Multilocus Sequence Typing for Epidemiological and Evolutionary Analyses of Phytopathogenic Xanthomonas citri

**DOI:** 10.1128/aem.02101-22

**Published:** 2023-04-17

**Authors:** R. Ragupathy, K. A. Jolley, C. Zamuner, J. B. Jones, J. Redfern, F. Behlau, H. Ferreira, M. C. Enright

**Affiliations:** a Department of Life Sciences, Manchester Metropolitan University, Manchester, United Kingdom; b Department of Biology, University of Oxford, Oxford, United Kingdom; c Departamento de Biologia Geral e Aplicada, Universidade Estadual Paulista, Rio Claro, São Paulo, Brazil; d Department of Plant Pathology, University of Florida, Gainesville, Florida, USA; e Fundecitrus, Araraquara, São Paulo, Brazil; INRS Armand-Frappier Sante Biotechnologie Research Centre

**Keywords:** citrus canker, MLST, *Xanthomonas citri*, cgMLST

## Abstract

Xanthomonas citri subsp. *citri* is the cause of bacterial citrus canker, responsible for major economic losses to the citrus industry. X. citri subspecies and pathovars are responsible for diseases in soybean, common bean, mango, pomegranate, and cashew. *X. citri* disease has been tracked using several typing methods, but recent studies using genomic sequencing have been key to understanding the evolutionary relationships within the species, including fundamental differences among *X. citri* subsp. *citri* pathotypes. Here, we describe a core-genome multilocus sequence typing (cgMLST) scheme for *X. citri* based on 250 genomes comprising multiple examples of *X. citri* subsp. *citri* pathotypes A, A*, and A^w^; *X. citri* subsp. *malvacearum*; *X. citri* pv. *aurantifolii*, pv. *fuscans*, pv. *glycines*, pv. *mangiferaeindicae*, pv. *viticola*, and pv. *vignicola*; and single isolates of *X. citri* pv. *dieffenbachiae* and pv. *punicae*. This data set included genomic sequencing of 100 novel *X. citri* subsp. *citri* isolates. cgMLST, based on 1,618 core genes across 250 genomes, is implemented at PubMLST (https://pubmlst.org/organisms/xanthomonas-citri/). GrapeTree minimum-spanning tree and Interactive Tree of Life (iTOL) neighbor-joining phylogenies generated from the cgMLST data resolved almost identical groupings of isolates to a core-genome single nucleotide polymorphism (SNP)-based neighbor-joining phylogeny. These resolved identical groupings of *X. citri* subsp. *citri* pathotypes and *X. citri* subspecies and pathovars. *X. citri* cgMLST should prove to be an increasingly valuable resource for the study of this key species of plant-pathogenic bacteria. Users can submit genomic data and associated metadata for comparison with previously characterized isolates at PubMLST to allow the rapid characterization of the local, national, and global epidemiology of these pathogens and examine evolutionary relationships.

**IMPORTANCE** Xanthomonas citri is a plant pathogen that causes major economic losses to the citrus industry and sweet orange production in particular. Several subspecies and pathogens are recognized, with host ranges including soybean, common bean, mango, pomegranate, and cashew, among others. Recent genomic studies have shown that host-adapted *X. citri* subspecies and pathovars and *X. citri* subsp. *citri* pathotypes form distinct clades. In this study, we describe a core-genome multilocus sequence typing (cgMLST) scheme for this species that can rapidly and robustly discriminate among these ecologically distinct, host-adapted clades. We have established this scheme and associated databases containing genomic sequences and metadata at PubMLST, which users can interrogate with their own genome sequences to determine *X. citri* subspecies, pathovars, and pathotypes. *X. citri* cgMLST should prove to be an invaluable tool for the study of the epidemiology and evolution of this major plant pathogen.

## INTRODUCTION

Bacterial citrus canker has a major economic impact on the production of all commercial citrus crops, including oranges, limes, tangerines, lemons, and grapefruit. Three pathotypes of canker are recognized: A, B, and C. Type A, caused by Xanthomonas citri subsp. *citri*, is the most widespread and economically damaging, whereas types B and C, caused by *X. citri* pv. *aurantifolii*, have much-reduced virulence on sweet orange and have very limited geographical spread (1). The type A (2, 3) pathotype has the broadest host range and infects most economically important citrus plants worldwide, particularly causing a major economic burden on the South American and Californian orange industries (2, 4). Two variants of pathotype A have evolved: A*, which can cause canker on all citrus but with some isolates that can infect only key lime (Citrus aurantifolia), and A^w^, which infects only key lime and alemow (Citrus macrophylla) (1).

Xanthomonas citri subspecies and pathovars other than *X. citri* subsp. *citri* infect other important crop species, including common bean (X. citri pv. *fuscans*), Mexican lime (X. citri pv. *aurantifolii*), mango (X. citri pv. *mangiferaeindicae*), grape (X. citri pv. *viticola*), cotton (X. citri subsp. *malvacearum*), soybean (X. citri pv. *glycines*), Araceae (X. citri pv. *dieffenbachiae*), cashew (X. citri pv. *anacardii*), and pomegranate (X. citri pv. *punicae*). Previous genome sequencing studies have examined the evolution of *X. citri* pathovars and subspecies (5) and *X. citri* subsp. *citri* pathotypes (4), and these studies have produced robust phylogenies that clearly resolve clades corresponding to individual *X. citri* pathovars and *X. citri* subsp. *citri* pathotypes. Genomic sequencing has also proven useful in investigations of host-pathogen interactions through the identification of host-specific virulence factors (6).

Whole-genome sequencing has greatly advanced the study of the epidemiology and evolution of pathogenic bacteria, greatly improving the discriminatory power and portability of other approaches such as ribotyping or pulsed-field gel electrophoresis (7). Genomic sequencing and analysis tools, developed primarily for the study of human bacterial pathogens to track and investigate outbreaks of disease caused by particularly virulent or antimicrobial-resistant clones, can also be usefully employed for the study of bacterial plant disease epidemiology and evolution.

Whole-genome sequences from isolates of pathogenic bacteria are usually compared using SNP (single nucleotide polymorphism)-based approaches that involve whole-genome alignments. Such SNP-based approaches have been used in recent studies of Xanthomonas citri biology (8, 9); however, they involve identifying genomes of isolates from the literature and downloading their sequences, followed by the computationally intensive alignment of multiple genomes to generate SNP profiles, which are then used to produce phylogenetic trees using methods such as neighbor joining (NJ), maximum parsimony, or maximum likelihood. Core-genome multilocus sequence typing (cgMLST) uses whole-genome sequence data to examine genetic similarities between isolates. It is based on allelic variations at a large number of core-genome loci that are present in all, or nearly all, members of a species (10). It differs from other whole-genome sequencing approaches in that it does not include noncore, accessory genes in comparisons of genomes, and it examines variation in allelic profiles rather than core-genome SNPs. In addition, cgMLST is computationally efficient, scalable, and suited for the representation of very large numbers of genomic comparisons. cgMLST schemes have been established for a diverse range of human pathogens, and some schemes contain many thousands of genomes. For example, the curated, open-source database PubMLST (https://pubmlst.org/) contains genomic data and metadata for 655,340 genomes of >100 bacterial species, and the EnteroBase database (https://enterobase.warwick.ac.uk) contains 379,370 Salmonella and 237,066 Escherichia coli/*Shigella* genomes and corresponding metadata alone (as of 23 November 2022).

In this study, we describe a cgMLST scheme and website resource that can be used to rapidly and easily identify *X. citri* subsp. *citri* variants from genome sequences without the need for computationally intensive and time-consuming core-genome SNP extraction, genome alignment, and phylogenetic comparisons. The *X. citri* cgMLST database at https://pubmlst.org/organisms/xanthomonas-citri represents an invaluable resource for tracking the spread of pathovars of this devastating pathogen, which should also prove to be a useful, scalable tool in future national and international efforts to control citrus canker and other crop diseases.

## RESULTS

### Genome sequencing.

Assemblies from each *X. citri* isolate consisted of between 61 and 161 contigs, with *N*_50_ values of between 96,324 and 1,044,915 nucleotides (nt) and an average depth of coverage of 102× (range = 31× to 900×). For all isolates, more than 99% of reads were mapped to the family *Xanthomonadaceae* using Kraken (11).

### rMLST.

**FIG 1:**
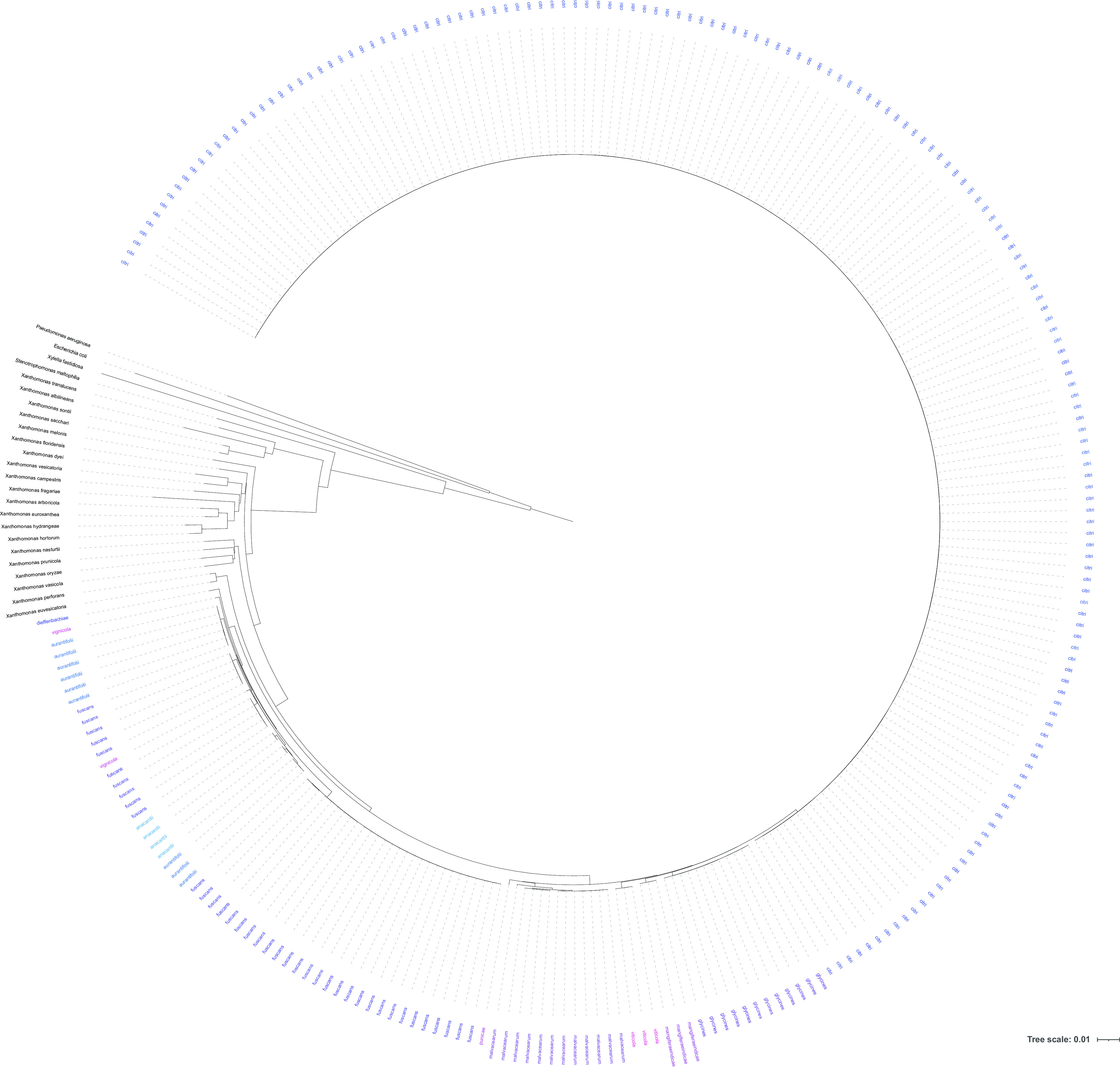
Neighbor-joining tree based on 43 concatenated rRNA gene sequences generated on the PubMLST website. The phylogeny was generated using the iTOL (24) plug-in on the PubMLST website (https://pubmlst.org/). The scale bar represents the genetic distance.

Ribosomal MLST (rMLST) confirmed the species designations of the 250 *X*. *citri* isolates listed in Table 1 as well as 20 other *Xanthomonas* spp. and the 4 other species examined and listed in Table S1 in the supplemental material. Figure 1 shows a neighbor-joining tree of all 274 genomes in this study based on the 53 concatenated rRNA gene loci used in the rMLST scheme. It can be clearly seen that all *X. citri* isolates form a separate and distinct clade whose closest neighbors are genomes of other *X. citri* pathotypes and subspecies. From this analysis, the *Xanthomonas* species X. vasicola and X. perforans appear to be the most closely related to *X. citri*, with the genomes of other xanthomonads such as X. euvesicatoria being separated by greater genetic distances. Example genomes of E. coli, Pseudomonas aeruginosa, Xylella fastidiosa, and Stenotrophomonas maltophilia are separated by even larger genetic distances from the genomes of *Xanthomonas* spp., including *X. citri.*

**TABLE 1:** Details of isolates and genomes used in this study^*a*^

MLST ID	Isolate	Alias	Country	Region	Yr of isolation	Source	Plant host species	*X. citri* pathovar or subspecies	Pathotype	BioProject accession no.	BioSample accession no.	Reference	cgMLST group (≤200 mismatches)
1	306	IBSBF 1594	Brazil	Paraná		Leaf	Sweet orange	*citri*	A	PRJNA779375	NA	14	1
2	FDC102		Brazil			Leaf	Sweet orange	*citri*	A	PRJNA779375	SAMN23028269	This study	1
3	FDC103		Argentina	Corrientes		Leaf	Sweet orange	*citri*	A	PRJNA779375	SAMN23028270	This study	1
4	FDC104		Paraguay			Leaf	Sweet orange	*citri*	A	PRJNA779375	SAMN23028271	This study	1
5	FDC1053		Brazil	Ilha Solteira, São Paulo	2004	Leaf	Sweet orange	*citri*	A	PRJNA779375	SAMN23028272	This study	1
6	FDC107		Uruguay	Salto		Leaf	Sweet orange	*citri*	A	PRJNA779375	SAMN23028273	This study	1
7	FDC1083	IBSBF 256	Brazil	Assis, São Paulo	1980	Leaf	Sweet orange	*citri*	A	PRJNA779375	SAMN23028274	This study	1
8	FDC1085	IBSBF 314	Brazil	Araçatuba, São Paulo	1979	Leaf	Sweet orange	*citri*	A	PRJNA779375	SAMN23028275	This study	1
9	FDC1087	IBSBF 338	Brazil	Cândido Mota, São Paulo	1981	Leaf	Sweet orange	*citri*	A	PRJNA779375	SAMN23028276	This study	1
10	FDC1088	IBSBF 340	Brazil	Lins, São Paulo	1981	Leaf	Sweet orange	*citri*	A	PRJNA779375	SAMN23028277	This study	1
11	FDC1091	IBSBF 353	Brazil	São Pedro do Turvo, São Paulo	1981	Leaf	Sweet orange	*citri*	A	PRJNA779375	SAMN23028278	This study	1
12	FDC1094	IBSBF 438	Brazil	Cajobi, São Paulo	1982	Leaf	Sweet orange	*citri*	A	PRJNA779375	SAMN23028279	This study	1
13	FDC1095	IBSBF 491	Brazil	Santa Mercedes, São Paulo	1983	Leaf	Sweet orange	*citri*	A	PRJNA779375	SAMN23028280	This study	1
14	FDC1098	IBSBF 947	Brazil	Presidente Prudente, São Paulo	1992	Leaf	Sweet orange	*citri*	A	PRJNA779375	SAMN23028281	This study	1
15	FDC1101	IBSBF 1287	Brazil	Moji-Mirim, São Paulo	1996	Leaf	Sweet orange	*citri*	A	PRJNA779375	SAMN23028282	This study	1
16	FDC1102	IBSBF 1403	Brazil	São João da Boa Vista, São Paulo	1998	Leaf	Sweet orange	*citri*	A	PRJNA779375	SAMN23028283	This study	1
17	FDC1104	IBSBF 1415	Brazil	Engenheiro Coelho, São Paulo	1998	Leaf	Sweet orange	*citri*	A	PRJNA779375	SAMN23028284	This study	1
18	FDC1107	IBSBF 1428	Brazil	Itirapina, São Paulo	1999	Leaf	Sweet orange	*citri*	A	PRJNA779375	SAMN23028285	This study	1
19	FDC1115	IBSBF 1440	Brazil	General Salgado, São Paulo	1999	Leaf	Sweet orange	*citri*	A	PRJNA779375	SAMN23028286	This study	1
20	FDC1116	IBSBF 1449	Brazil	Presidente Bernardes, São Paulo	1999	Leaf	Sweet orange	*citri*	A	PRJNA779375	SAMN23028287	This study	1
21	FDC1118	IBSBF 1453	Brazil	Botucatu, São Paulo	1999	Leaf	Sweet orange	*citri*	A	PRJNA779375	SAMN23028288	This study	1
22	FDC1120	IBSBF 1484	Brazil	Clementina, São Paulo	2000	Leaf	Sweet orange	*citri*	A	PRJNA779375	SAMN23028289	This study	1
23	FDC1121	IBSBF 1485	Brazil	Luiziânia, São Paulo	2000	Leaf	Sweet orange	*citri*	A	PRJNA779375	SAMN23028290	This study	1
24	FDC1125	IBSBF 1491	Brazil	Sud Mennucci, São Paulo	2000	Leaf	Sweet orange	*citri*	A	PRJNA779375	SAMN23028291	This study	1
25	FDC1129	IBSBF 1518	Brazil	Guzolândia, São Paulo	2000	Leaf	Sweet orange	*citri*	A	PRJNA779375	SAMN23028292	This study	1
26	FDC1139	IAPAR 12426	Brazil	Araraquara, São Paulo	1999	Leaf	Sweet orange	*citri*	A	PRJNA779375	SAMN23028293	This study	1
27	FDC1142		Brazil			Leaf	Sweet orange	*citri*	A	PRJNA779375	SAMN23028294	This study	1
28	FDC1143	IAPAR 12778	Brazil	Avaré, São Paulo	2000	Leaf	Sweet orange	*citri*	A	PRJNA779375	SAMN23028295	This study	1
29	FDC1144	IAPAR 12822	Brazil	Guaimbê, São Paulo	2000	Leaf	Sweet orange	*citri*	A	PRJNA779375	SAMN23028296	This study	1
30	FDC1145	IAPAR 12989	Brazil	Marília, São Paulo	2000	Leaf	Sweet orange	*citri*	A	PRJNA779375	SAMN23028297	This study	1
31	FDC1148	IAPAR 12991	Brazil	Tarumã, São Paulo	2001	Leaf	Sweet orange	*citri*	A	PRJNA779375	SAMN23028298	This study	1
32	FDC1150	IAPAR 12001	Brazil	Salto Grande, São Paulo	2001	Leaf	Sweet orange	*citri*	A	PRJNA779375	SAMN23028299	This study	1
33	FDC1182		Brazil	Ourizona, Paraná	2005	Leaf	Sweet orange	*citri*	A	PRJNA779375	SAMN23028300	This study	1
34	FDC122		China	Hong Kong		Leaf	Sweet orange	*citri*	A	PRJNA779375	SAMN23028301	This study	1
35	FDC1227		Brazil	Adolfo, São Paulo	2005	Leaf	Sweet orange	*citri*	A	PRJNA779375	SAMN23028302	This study	1
36	FDC124		Japan			Leaf	Sweet orange	*citri*	A	PRJNA779375	SAMN23028303	This study	1
37	FDC1252		Brazil	Sales, São Paulo	2005	Leaf	Sweet orange	*citri*	A	PRJNA779375	SAMN23028304	This study	1
38	FDC126		Philippines			Leaf	Sweet orange	*citri*	A	PRJNA779375	SAMN23028305	This study	1
39	FDC1277		Brazil	Marinópolis, São Paulo	2005	Leaf	Sweet orange	*citri*	A	PRJNA779375	SAMN23028306	This study	1
40	FDC129		France	Reunion		Leaf	Sweet orange	*citri*	A	PRJNA779375	SAMN23028307	This study	1
41	FDC1291		Brazil	Rubiácea, São Paulo	2007	Leaf	Sweet orange	*citri*	A	PRJNA779375	SAMN23028308	This study	1
42	FDC130		China			Leaf	Sweet orange	*citri*	A	PRJNA779375	SAMN23028309	This study	1
43	FDC131		Thailand			Leaf	Sweet orange	*citri*	A	PRJNA779375	SAMN23028310	This study	1
44	FDC133		Mauritius			Leaf	Sweet orange	*citri*	A	PRJNA779375	SAMN23028311	This study	1
45	FDC1387		Brazil	Urânia, São Paulo	2007	Leaf	Sweet orange	*citri*	A	PRJNA779375	SAMN23028312	This study	1
46	FDC1424		Brazil	Suzanápolis, São Paulo	2007	Leaf	Sweet orange	*citri*	A	PRJNA779375	SAMN23028313	This study	1
47	FDC1488		Brazil	Urânia, São Paulo	2007	Leaf	Sweet orange	*citri*	A	PRJNA779375	SAMN23028314	This study	1
48	FDC15		Argentina	Entre Rios		Leaf	Sweet orange	*citri*	A	PRJNA779375	SAMN23028315	This study	1
49	FDC1531		Brazil	Pereira Barreto, São Paulo	2007	Leaf	Sweet orange	*citri*	A	PRJNA779375	SAMN23028316	This study	1
50	FDC1533		Brazil	Palmeira d’Oeste, São Paulo	2007	Leaf	Sweet orange	*citri*	A	PRJNA779375	SAMN23028317	This study	1
51	FDC1539		Brazil	Ilha Solteira, São Paulo	2007	Leaf	Sweet orange	*citri*	A	PRJNA779375	SAMN23028318	This study	1
52	FDC1580		Brazil	Boa Esperança do Sul, São Paulo	2007	Leaf	Sweet orange	*citri*	A	PRJNA779375	SAMN23028319	This study	1
53	FDC1666		Brazil	Rondon, Paraná	2011	Leaf	Sweet orange	*citri*	A	PRJNA779375	SAMN23028320	This study	1
54	FDC1681		Brazil	Matão, São Paulo	2012	Leaf	Sweet orange	*citri*	A	PRJNA779375	SAMN23028321	This study	1
55	FDC1705		Brazil	Paranavaí, Paraná	2013	Leaf	Sweet orange	*citri*	A	PRJNA779375	SAMN23028322	This study	1
56	FDC1707		Brazil	Alto Paraná, Paraná	2013	Leaf	Sweet orange	*citri*	A	PRJNA779375	SAMN23028323	This study	1
57	FDC1733		Brazil	Guairaçã, Paraná	2014	Leaf	Sweet orange	*citri*	A	PRJNA779375	SAMN23028324	This study	1
58	FDC2		Brazil	Lins, São Paulo	2001	Leaf	Sweet orange	*citri*	A	PRJNA779375	SAMN23028325	This study	1
59	FDC24		Paraguay			Leaf	Sweet orange	*citri*	A	PRJNA779375	SAMN23028326	This study	1
60	FDC4167		Unknown			Leaf	Sweet orange	*citri*	A	PRJNA779375	SAMN23028327	This study	1
61	FDC46		New Zealand	New Plymouth		Leaf	Sweet orange	*citri*	A	PRJNA779375	SAMN23028328	This study	1
62	FDC49		Japan			Leaf	Sweet orange	*citri*	A	PRJNA779375	SAMN23028329	This study	1
63	FDC50		Fiji	Vanua Levu		Leaf	Sweet orange	*citri*	A*	PRJNA779375	SAMN23028330	This study	2
64	FDC502		New Zealand			Leaf	Sweet orange	*citri*	A	PRJNA779375	SAMN23028331	This study	1
65	FDC512		Brazil	Iacri, São Paulo	2002	Leaf	Sweet orange	*citri*	A	PRJNA779375	SAMN23028332	This study	1
66	FDC52		India	New Delhi		Leaf	Sweet orange	*citri*	A	PRJNA779375	SAMN23028333	This study	1
67	FDC53		Iran			Leaf	Sweet orange	*citri*	A*	PRJNA779375	SAMN23028334	This study	2
68	FDC54		Australia	Darwin		Leaf	Sweet orange	*citri*	A	PRJNA779375	SAMN23028335	This study	1
69	FDC544		Brazil	Mira Estrela, São Paulo	2002	Leaf	Sweet orange	*citri*	A	PRJNA779375	SAMN23028336	This study	1
70	FDC55		Taiwan			Leaf	Sweet orange	*citri*	A	PRJNA779375	SAMN23028337	This study	1
71	FDC550		Brazil	Rubinéia, São Paulo	2002	Leaf	Sweet orange	*citri*	A	PRJNA779375	SAMN23028338	This study	1
72	FDC551		Brazil	Ibitinga, São Paulo	2002	Leaf	Sweet orange	*citri*	A	PRJNA779375	SAMN23028339	This study	1
73	FDC553		Brazil	Avaré, São Paulo	2001	Leaf	Sweet orange	*citri*	A	PRJNA779375	SAMN23028340	This study	1
74	FDC559		Brazil	Cafelândia, São Paulo		Leaf	Sweet orange	*citri*	A	PRJNA779375	SAMN23028341	This study	1
75	FDC560		Brazil	Urupês, São Paulo	2001	Leaf	Sweet orange	*citri*	A	PRJNA779375	SAMN23028342	This study	1
76	FDC562		Brazil	Terra Roxa	2002	Leaf	Sweet orange	*citri*	A	PRJNA779375	SAMN23028343	This study	1
77	FDC565		Brazil	Barbosa, São Paulo	2002	Leaf	Sweet orange	*citri*	A	PRJNA779375	SAMN23028344	This study	1
78	FDC575		Brazil	Barbosa, São Paulo	2002	Leaf	Sweet orange	*citri*	A	PRJNA779375	SAMN23028345	This study	1
79	FDC601	IBSBF 1989	Brazil	Rio Grande do Sul	2002	Leaf	Sweet orange	*citri*	A	PRJNA779375	SAMN23028346	This study	1
80	FDC603	IBSBF 1990	Brazil	Mariana Moro, Rio Grande do Sul		Leaf	Sweet orange	*citri*	A	PRJNA779375	SAMN23028347	This study	1
81	FDC7		Brazil	Bataguassu, Mato Grosso do Sul		Leaf	Sweet orange	*citri*	A	PRJNA779375	SAMN23028348	This study	1
82	FDC704		Brazil	Nova Canaã, São Paulo	2002	Leaf	Sweet orange	*citri*	A	PRJNA779375	SAMN23028349	This study	1
83	FDC705		Brazil	Nova Canaã, São Paulo	2002	Leaf	Sweet orange	*citri*	A	PRJNA779375	SAMN23028350	This study	1
84	FDC714		Brazil	Jales, São Paulo	2002	Leaf	Sweet orange	*citri*	A	PRJNA779375	SAMN23028351	This study	1
85	FDC718		Brazil	Borborema, São Paulo	2002	Leaf	Sweet orange	*citri*	A	PRJNA779375	SAMN23028352	This study	1
86	FDC719		Brazil	Parapuã, São Paulo	2002	Leaf	Sweet orange	*citri*	A	PRJNA779375	SAMN23028353	This study	1
87	FDC724		Brazil	Urupês, São Paulo	2002	Leaf	Sweet orange	*citri*	A	PRJNA779375	SAMN23028354	This study	1
88	FDC748		Brazil	Osvaldo Cruz, São Paulo	2002	Leaf	Sweet orange	*citri*	A	PRJNA779375	SAMN23028355	This study	1
89	FDC749		Brazil	Aparecida do Oeste, São Paulo	2002	Leaf	Sweet orange	*citri*	A	PRJNA779375	SAMN23028356	This study	1
90	FDC75	ISBSF 1421	Brazil	Casa Branca, São Paulo	1998	Leaf	Sweet orange	*citri*	A	PRJNA779375	SAMN23028357	This study	1
91	FDC755		Brazil	Caiuá, São Paulo	2002	Leaf	Sweet orange	*citri*	A	PRJNA779375	SAMN23028358	This study	1
92	FDC764		Brazil	Sandovalina, São Paulo	2002	Leaf	Sweet orange	*citri*	A	PRJNA779375	SAMN23028359	This study	1
93	FDC769		Brazil	Narandiba, São Paulo	2002	Leaf	Sweet orange	*citri*	A	PRJNA779375	SAMN23028360	This study	1
94	FDC782		Brazil	Ibitinga, São Paulo	2002	Leaf	Sweet orange	*citri*	A	PRJNA779375	SAMN23028361	This study	1
95	FDC8		Brazil	Corbélia, Paraná		Leaf	Sweet orange	*citri*	A	PRJNA779375	SAMN23028362	This study	1
96	FDC806		Brazil	Boa Vista, Roraima	2002	Leaf	Sweet orange	*citri*	A	PRJNA779375	SAMN23028363	This study	1
97	FL71		USA	Florida		Leaf	Sweet orange	*citri*	A	PRJNA779375	SAMN23028364	This study	1
98	FL72		USA	Florida		Leaf	Sweet orange	*citri*	A	PRJNA779375	SAMN23028365	This study	1
99	FL75		USA			Leaf	Sweet orange	*citri*	A	PRJNA779375	SAMN23028366	This study	1
100	LM199		Argentina		2015	Leaf	Sweet orange	*citri*	A	PRJNA779375	SAMN23028367	This study	1
101	FDC4		Brazil	Cuiabá-Mato Grosso	2001	Leaf	Sweet orange	*citri*	A	PRJNA779375	SAMN23028368	This study	1
102	12_2		Thailand	Nakhon Ratchasima	1991	Leaf	Soybean	*glycines*		PRJNA323439	SAMN05179543		3
103	1017		South Korea	Suwon	1997	Unknown		*glycines*		PRJNA556098	SAMN12340733		3
104	1018		South Korea	Hwaseong	1997	Unknown		*glycines*		PRJNA556099	SAMN12340735		3
105	1045		South Korea	Hwaseong	1997	Unknown		*glycines*		PRJNA556109	SAMN12340804		3
106	1157		South Korea	Pocheon	1997	Unknown		*glycines*		PRJNA556102	SAMN12340780		3
107	1566		Brazil			Unknown		*aurantifolii*		PRJNA273983	SAMN03835495		4
108	4834-R		France	Beaucouzé	1998	Unknown	Common bean	*fuscans*		PRJNA176873	SAMEA3283112	25	5
109	5208		USA	Florida	2002	Unknown		*citri*		PRJNA255042	SAMN02911840	26	1
110	12609		Taiwan		2015	Leaf		*glycines*		PRJNA344018	SAMN05818161		3
112	AR81009		Argentina		1981	Unknown		*malvacearum*		PRJNA396899	SAMN07447516		6
113	AS8		Saudi Arabia			Unknown		*citri*	A*	PRJNA255042	SAMN02911853	26	2
114	AS9		Saudi Arabia			Unknown		*citri*	A*	PRJNA255042	SAMN02911854	26	2
115	AS270		Saudi Arabia		1988	Unknown		*citri*	A*	PRJNA255042	SAMN02911852	26	2
116	AW13		USA	Florida	2003	Unknown		*citri*	A^w^	PRJNA255042	SAMN02911848	26	7
117	AW14		USA	Florida	2005	Unknown		*citri*	A^w^	PRJNA255042	SAMN02911849	26	7
118	AW15		USA	Florida	2005	Unknown		*citri*	A^w^	PRJNA255042	SAMN02911850	26	7
119	AW16		USA	Florida	2005	Unknown		*citri*	A^w^	PRJNA255042	SAMN02911851	26	7
120	Aw12879		USA	Florida		Unknown		*citri*	A^w^	PRJNA81931	SAMN02603165	27	7
121	BL18		USA	Florida	2011	Unknown		*citri*		PRJNA255042	SAMN02911845	26	1
122	CCRMXCV-80		Brazil	Pernambuco	2015	Unknown		*viticola*		PRJNA407795	SAMN07664206		8
123	CFBP6988		Réunion		2000	Unknown		*fuscans*		PRJNA212252	SAMN02645882	28	9
124	CFBP1815		Greece		1978	Unknown	Common bean	*fuscans*		PRJEB23080	SAMEA104357164	28	5
125	CFBP2526		Sudan		1956	Unknown	Soybean	*glycines*		PRJNA212247	SAMN02469936	29	3
126	CFBP2913		Brazil		1974	Unknown		*anacardii*		PRJNA232107	SAMN07766623	30	10
127	CFBP4884		France		1998	Leaf		*fuscans*		PRJNA254240	SAMN02902416	31	5
128	CFBP4885		France		1998	Unknown		*fuscans*		PRJNA384182	SAMN06829861	28	5
129	CFBP6165		Canada		1957	Unknown		*fuscans*		PRJNA384145	SAMN06829306	28	11
130	CFBP6166		South Africa		1963	Unknown		*fuscans*		PRJNA384183	SAMN06829862	28	5
131	CFBP6167		USA		1954	Unknown		*fuscans*		PRJNA384187	SAMN06829865	28	5
132	CFBP6960		Réunion		2000	Unknown	Common bean	*fuscans*		PRJEB23080	SAMEA104357193	28	5
133	CFBP6970		USA		1990	Unknown	Common bean	*fuscans*		PRJEB23080	SAMEA104357194	28	5
134	CFBP6975		France		1994	Unknown		*fuscans*		PRJNA384188	SAMN06829867	28	5
135	CFBP6988R		Réunion		2000	Unknown		*fuscans*		PRJNA384160	SAMN06829542	28	9
136	CFBP6989		Réunion		2000	Unknown		*fuscans*		PRJNA384161	SAMN06829585	28	9
137	CFBP6990		Réunion		2000	Unknown		*fuscans*		PRJNA384163	SAMN06829587	28	9
138	CFBP6991		Réunion		2000	Unknown		*fuscans*		PRJNA384178	SAMN06829858	28	9
139	CFBP6992		Réunion		2000	Unknown		*fuscans*		PRJNA384177	SAMN06829854	28	13
140	CFBP6994		Tanzania		1990	Unknown	Common bean	*fuscans*		PRJEB23080	SAMEA104357310	28	Singleton
141	CFBP6994R		Tanzania		1990	Unknown		*fuscans*		PRJNA384179	SAMN06829859	28	13
142	CFBP6996		Réunion		2000	Leaf	Common bean	*fuscans*		PRJNA212255	SAMN02645883		13
143	CFBP6996R		Réunion		2000	Unknown		*fuscans*		PRJNA384180	SAMN06829860		13
144	CFBP7111		USA	Texas	1942	Leaf		*vignicola*		PRJNA390891	SAMN07252112		Singleton
145	CFBP7113		Sudan		1966	Unknown		*vignicola*		PRJNA390890	SAMN07251989		Singleton
146	CFBP7119		Brazil		1981	Unknown	Soybean	*glycines*		PRJNA212249	SAMN02469937	29	3
147	CFBP7764		Brazil	Petrolina	2012	Stem		*viticola*		PRJNA422087	SAMN08163769		8
148	CFBP7766		Cameroon		2009	Unknown	Common bean	*fuscans*		PRJEB23080	SAMEA104357197	28	5
149	CFBP7767		Cameroon		2009	Unknown	Common bean	*fuscans*		PRJEB23080	SAMEA104357198	28	5
150	EB08		USA	Central Iowa	2008	Unknown		*glycines*		PRJNA431457	SAMN08391414	32	3
151	FB19		USA	Florida	2011	Unknown		*citri*		PRJNA255042	SAMN02911846	26	1
152	FDC535		Brazil	Sao Paulo	2000	Unknown		*aurantifolii*		PRJNA273983	SAMN03317023		Singleton
153	FDC628		Brazil	Santa Catarina	2001	Unknown		*citri*		PRJNA273983	SAMN03317028		1
154	FDC636		Brazil	Paraná	1996	Unknown		*citri*		PRJNA273983	SAMN03317019		1
155	FDC654		Brazil	Rio Grande do Sul	1999	Unknown		*citri*		PRJNA273983	SAMN03317029		1
156	FDC763		Brazil	São Paulo	1981	Unknown		*aurantifolii*		PRJNA273983	SAMN03317030		17
157	FDC828		Brazil	São Paulo	1997	Unknown		*citri*		PRJNA273983	SAMN03317018		1
158	FDC867		Brazil	São Paulo	2002	Unknown		*aurantifolii*		PRJNA273983	SAMN03317026		17
159	FDC1559		Brazil	São Paulo	1981	Unknown		*aurantifolii*		PRJNA273983	SAMN03317020		17
160	FDC1561		Argentina		1981	Unknown		*aurantifolii*		PRJNA273983	SAMN03317022		4
161	FDC1609		Brazil	Sao Paulo	2009	Unknown		*aurantifolii*		PRJNA273983	SAMN03317027		Singleton
162	FDC1662		Brazil	Paraná	2011	Unknown		*citri*		PRJNA273983	SAMN03317017		1
163	FDC1682		Oman		1986	Unknown		*citri*	A*	PRJNA273983	SAMN03317024		2
164	GD2		China	Guangdong	2011	Unknown		*citri*		PRJNA255042	SAMN02911834	26	1
165	GD3		China	Guangdong	2011	Unknown		*citri*		PRJNA255042	SAMN02911835	26	1
166	GSPB1386		Nicaragua		1986	Unknown		*malvacearum*		PRJNA78127	SAMN02469610		18
167	GSPB2388		Sudan			Unknown		*malvacearum*		PRJNA79081	SAMN02469611		Singleton
168	HD-1		China			Leaf		*malvacearum*		PRJNA587534	SAMN13193097		6
169	IBSBF2579		Brazil		2009	Unknown		*anacardii*		PRJNA416784	SAMN07964563		10
170	ICPB10535		Brazil			Unknown	Mexican lime	*aurantifolii*		PRJNA18835	SAMN02472096	33	Singleton
171	ICPB11122		Argentina			Unknown		*aurantifolii*		PRJNA18837	SAMN02472095	33	Singleton
172	ISO12C3		Canada	Ontario		Unknown	Common bean	*fuscans*		PRJNA289080	SAMN03842216		5
173	ISO118C1		Canada	Ontario		Unknown	Common bean	*fuscans*		PRJNA289080	SAMN03842217		5
174	ISO118C5		Canada	Ontario		Unknown	Common bean	*fuscans*		PRJNA289080	SAMN03842218		5
175	JJ10-1		Mauritius	Rodrigues Island	1985	Unknown		*citri*	A	PRJEB7180	SAMEA2844848	4	1
176	JK4-1		China		1985	Unknown		*citri*	A	PRJEB7186	SAMEA2844844	4	1
177	JK48		Saudi Arabia		1988	Unknown		*citri*	A*	PRJEB7195	SAMEA2827235	4	Singleton
178	JK143-9		Thailand		1990	Unknown		*citri*	A*	PRJEB7200	SAMEA2827230	4	Singleton
179	JK143-11		Thailand		1990	Unknown		*citri*	A*	PRJEB7184	SAMEA2844846	4	2
180	JM35-2		Saudi Arabia		1992	Unknown		*citri*	A*	PRJEB7189	SAMEA2827561	4	2
181	JS581		Iran		1997	Unknown		*citri*	A*	PRJEB7190	SAMEA2827560	4	2
182	JS582		Iran		1997	Unknown		*citri*	A*	PRJEB7203	SAMEA2827226	4	Singleton
183	jx-6		China	Jiangxi	2014	Unknown		*citri*		PRJNA286060	SAMN03765509		1
184	JX4		China	Jiangxi	2011	Unknown		*citri*		PRJNA255042	SAMN02911836	26	1
185	JX5		China	Jiangxi	2011	Unknown		*citri*		PRJNA255042	SAMN02911837	26	1
186	K2		South Korea	Danyang	2017	Unknown		*glycines*		PRJNA556107	SAMN12340803		3
187	LB100-1		Seychelles		2005	Unknown		*citri*	A	PRJEB7185	SAMEA2844845	4	1
188	LB302		USA	Florida	2002	Unknown		*citri*	A^w^	PRJEB7197	SAMEA2827233	4	Singleton
189	LE3-1		Ethiopia		2008	Unknown		*citri*	A*	PRJEB7194	SAMEA2827236	4	Singleton
190	LE116-1		Mali	Key lime	2008	Unknown		*citri*	A	PRJEB7201	SAMEA2827228	4	Singleton
191	LG56-10		Réunion		2009	Unknown	Mango	*mangiferaeindicae*		PRJNA232105	SAMN07766891		20
192	LG81-27		Réunion		2009	Unknown	Mango	*mangiferaeindicae*		PRJNA232105	SAMN07766892		20
193	LG97		Bangladesh		2006	Unknown		*citri*	A	PRJEB7196	SAMEA2827234	4	Singleton
194	LG98		Bangladesh		2006	Unknown		*citri*	A	PRJEB7183	SAMEA2844847	4	1
195	LG102		Bangladesh		2006	Unknown		*citri*	A	PRJEB7198	SAMEA2827232	4	Singleton
196	LG115		India		2007	Unknown		*citri*	A^w^	PRJEB7187	SAMEA2827563	4	7
197	LG117		Bangladesh		2009	Unknown		*citri*	A	PRJEB7188	SAMEA2827562	4	1
198	LH37-1		Senegal		2010	Unknown	Grapefruit	*citri*	A	PRJEB7192	SAMEA2827558	4	21
199	LH201		Réunion		2010	Leaf		*citri*		PRJNA344031	SAMN05823148		1
200	LH276		Réunion		2010	Leaf		*citri*		PRJNA344031	SAMN05823145		1
201	LJ207-7		Réunion		2012	Leaf		*citri*		PRJNA344031	SAMN05823144		1
202	LL074-4		Martinique		2014	Leaf		*citri*		PRJNA344031	SAMN05823143		1
203	LM180		Argentina		2003	Leaf		*citri*		PRJNA344031	SAMN05823141		1
204	LMG941		India		1948	Unknown		*mangiferaeindicae*		PRJEA86101	SAMEA2272013	34	20
205	LMG965		India		1969	Unknown	Grape	*viticola*		PRJEB5493	SAMEA3139044	35	8
206	LMG712		Sudan		1956	Unknown		*glycines*		PRJNA298608	SAMN04145201		3
207	LMG761		Sudan		1958	Unknown		*malvacearum*		PRJNA298617	SAMN04145210		6
208	LMG826		Belgium	Merelbeke	2014	Unknown	Common bean	*fuscans*		PRJNA254368	SAMN02903172	13	Singleton
209	LMG859		India		1959	Unknown		*punicae*		PRJNA73081	SAMEA3138416	36	Singleton
210	LMG7399		Belgium	Merelbeke	2014	Unknown		*dieffenbachiae*		PRJNA254467	SAMN02903999		Singleton
211	LMG9322		USA	Florida	1915	Unknown		*citri*	A	PRJNA338819	SAMN05571463		Singleton
212	mf20		USA	Florida	2011	Unknown		*citri*		PRJNA255042	SAMN02911847	26	1
213	MN10		USA	Florida	2005	Unknown		*citri*		PRJNA255042	SAMN02911841	26	1
214	MN11		USA	Florida		Unknown		*citri*		PRJNA255042	SAMN02911842	26	1
215	MN12		USA	Florida	1997	Unknown		*citri*		PRJNA255042	SAMN02911843	26	1
216	MS14003		USA	Wilzone, MS	2014	Leaf		*malvacearum*		PRJNA396899	SAMN07447517		18
217	MSCT		USA	Mississippi	2011	Leaf		*malvacearum*		PRJNA299817	SAMN05595756		18
218	NCPPB1402		Uganda		1962	Unknown	Common bean	*fuscans*		PRJNA264810	SAMN03142810		5
219	NCPPB381		Canada		1957	Unknown	Common bean	*fuscans*		PRJNA264772	SAMN03142398		11
220	NCPPB670		Uganda		1958	Unknown	Common bean	*fuscans*		PRJNA263153	SAMN03097370		5
221	NCPPB1056		Ethiopia		1961	Unknown	Common bean	*fuscans*		PRJNA264809	SAMN03142809		5
222	NCPPB1058		Ethiopia		1961	Unknown	Common bean	*fuscans*		PRJNA264815	SAMN03142815		5
223	NCPPB1433		Hungary		1956	Unknown	Common bean	*fuscans*		PRJNA264773	SAMN03142399		5
224	NCPPB1654		South Africa		1963	Unknown	Common bean	*fuscans*		PRJNA264775	SAMN03142402		5
225	NCPPB2665		Italy		1973	Unknown	Common bean	*fuscans*		PRJNA264776	SAMN03142403		5
226	NCPPB3607		India		1988	Unknown		*citri*	A*	PRJEB7191	SAMEA2827559	4	2
227	NCPPB3610		India		1988	Unknown		*citri*	A	PRJEB7199	SAMEA2827231	4	Singleton
228	NCPPB3612		India	Key lime	1988	Unknown		*citri*	A	PRJEB7193	SAMEA2827237	4	21
230	NCPPB3660		Brazil		1975	Unknown	Common bean	*fuscans*		PRJNA264814	SAMN03142811	13	5
231	NIGEB-88		Iran	Hashtbandi	2009	Unknown		*citri*	A*	PRJNA283400	SAMN03649471		2
232	NIGEB-386		Iran	Nik Shahr	2009	Unknown		*citri*	A*	PRJNA261284	SAMN03070127		2
233	NT17		USA	Florida	2011	Unknown		*citri*		PRJNA255042	SAMN02911844	26	1
234	TAQ18		Brazil			Leaf		*anacardii*		PRJNA416788	SAMN07964753		24
235	TAQ13		Brazil		2009	Leaf		*anacardii*		PRJNA416789	SAMN07964775		24
236	TX160042		USA	Rancho Viejo, TX	2015	Leaf		*citri*	A^w^	PRJNA381640	SAMN06685652		7
237	TX160149		USA	Rancho Viejo, TX	2015	Leaf		*citri*	A^w^	PRJNA381640	SAMN06685654		Singleton
238	TX160197		USA	Rancho Viejo, TX	2015	Leaf		*citri*	A^w^	PRJNA381640	SAMN06685696		7
239	UI6		China	Guangxi	2011	Unknown		*citri*		PRJNA255042	SAMN02911838	26	1
240	UI7		China	Guangxi	2011	Unknown		*citri*		PRJNA255042	SAMN02911839	26	1
241	WHRI5232		Sudan		1959	Unknown		*malvacearum*		PRJNA438827	SAMN08729580	37	6
242	03-1638-1-1		Argentina		2003	Unknown		*citri*		PRJNA401937	SAMN07611881	38	1
243	x8ra		South Korea	Suwon	1999	Unknown		*glycines*		PRJNA556081	SAMN12340633		3
244	X18		Burkina Faso			Unknown	Cotton	*malvacearum*		PRJNA172044	SAMN02469929	39	18
245	X20		Burkina Faso			Unknown	Cotton	*malvacearum*		PRJNA172045	SAMN02469930	39	6
246	X621		South Africa		1995	Unknown	Common bean	*fuscans*		PRJNA272380	SAMN03281080		5
247	Xcc29		China	Jiangxi	2010	Unknown		*citri*		PRJNA407058	SAMN07665076		1
248	Xcc49		China	Chongqing	2010	Unknown		*citri*		PRJNA407058	SAMN07638001		1
249	XcmH1005		USA	Oklahoma	1968	Unknown		*malvacearum*		PRJNA298765	SAMN04166563		6
250	XcmN1003		Burkina Faso		1967	Unknown		*malvacearum*		PRJNA298770	SAMN04166615		6
251	XCP631		Colombia	Quilichao	2004	Unknown	Common bean	*fuscans*		PRJNA272630	SAMN03284618		5
252	Xff49		Brazil	Pelotas	2017	Unknown		*fuscans*		PRJNA400313	SAMN07563171		Singleton

a NA, not applicable.

### cgMLST.

A total of 1,618 core genes (present in >99% of isolates) were found among 250 *X. citri* isolate genomes. These genes were numbered XCIT00001 to XCIT01618. Allele calling of the subset of the initial 100 records (from study isolates) resulted in isolates having between 99.4% and 100% of their loci with alleles designated. Core-genome MLST (cgMLST) groupings of the 250 genomes uploaded to the PubMLST website were made based on the number of allelic mismatches. This resulted in 171 groups of genomes with 5 or fewer mismatches (isolates tagged as Xc_cgc_5 on the PubMLST website), 113 with 10 or fewer mismatches (Xc_cgc_10), 53 with 50 or fewer mismatches (Xc_cgc_50), 39 with 100 or fewer mismatches (Xc_cgc_100), and 25 with 200 or fewer mismatches (Xc_cgc_200).

### cgMLST groupings.

The groupings of 250 isolates/genomes with fewer than 200 mismatches are shown in Table 1. Group 1 contained 132 *X. citri* subsp. *citri* isolates comprising 104 pathotype A isolates (pathotype data are missing for 28 isolates in this group); group 2 contained 12 *X. citri* subsp. *citri* genomes, all of which were isolates of pathotype A*; group 3 comprised 12 *X. citri* pv. *glycines* isolates; group 4 contained 2 *X. citri* pv. *aurantifolii* isolates; group 5 contained 24 *X. citri* pv. *fuscans* isolates; group 6 contained 7 *X. citri* subsp. *malvacearum* isolates; group 7 contained 8 *X. citri* pv. *citri* isolates with pathotype A^w^; group 8 contained 3 *X. citri* pv. *viticola* isolates; group 9 contained 5 *X. citri* pv. *fuscans* isolates; group 10 contained 2 *X. citri* pv. *anacardii* isolates; group 11 contained 2 *X. citri* pv. *fuscans* isolates; group 13 contained 4 *X. citri* pv. *fuscans* isolates; group 17 contained 3 *X. citri* pv. *aurantifolii* isolates; group 18 contained 4 *X. citri* subsp. *malvacearum* isolates; group 20 contained 3 *X. citri* pv. *mangiferaeindicae* isolates; group 21 contained 2 *X. citri* pv. *citri* isolates of an unknown pathotype; and group 24 contained 2 *X. citri* pv. *anacardii* isolates. Twenty-three isolates had no close matches using any of the allelic mismatch groupings described above, and these are referred to as singleton isolate genomes in Table 1. Other cgMLST groupings (and all other genomic data and metadata) can be found in Table 1.

### *X. citri* phylogeny.

**FIG 2:**
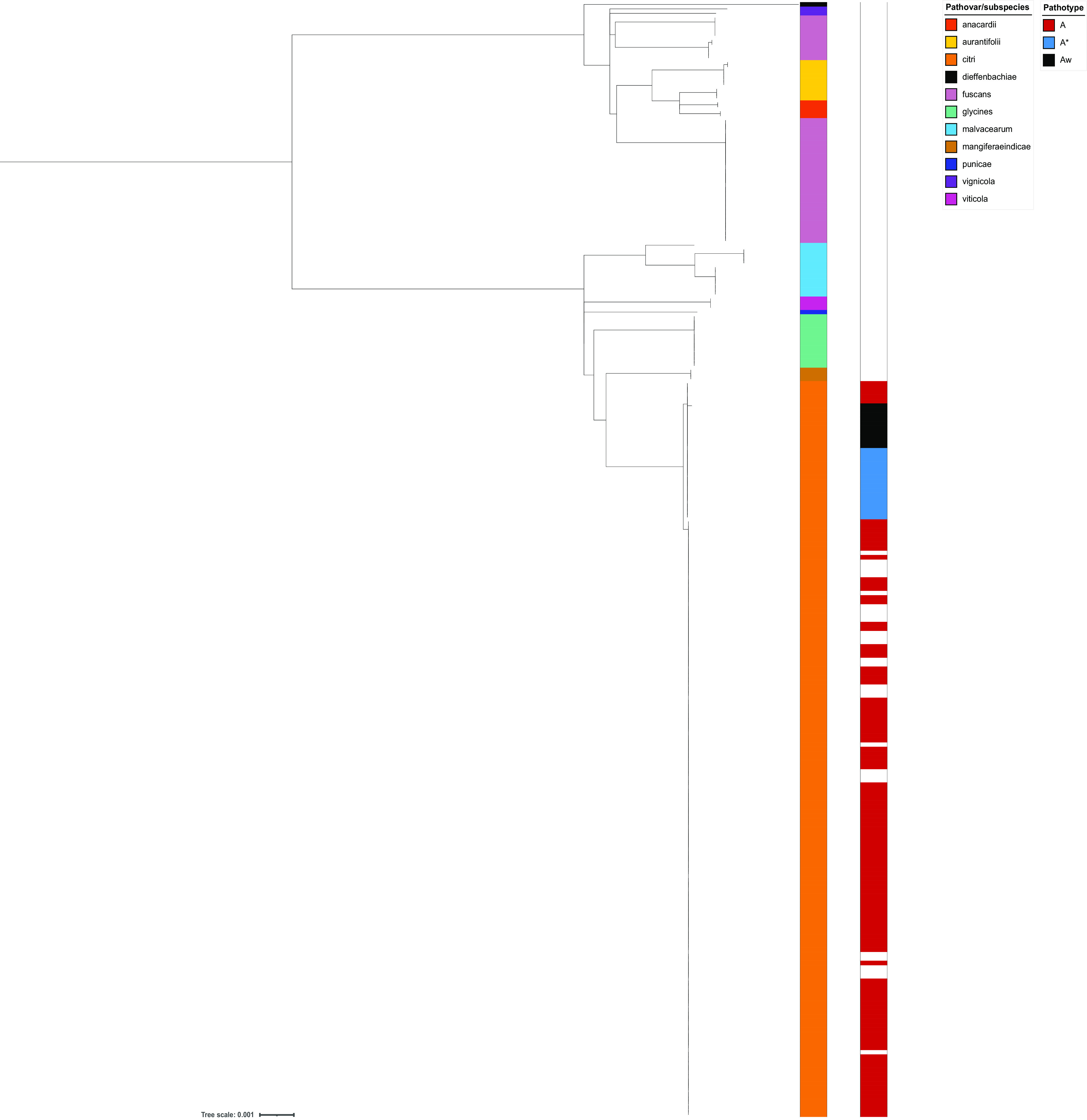
Neighbor-joining tree based on 250 concatenated core-genome MLST allele sequences of Xanthomonas citri. Isolates are colored according to their original pathovar/subspecies designations and *X. citri* subsp. *citri* pathotype. The phylogeny was generated using the iTOL (24) plug-in on the PubMLST website (https://pubmlst.org/). The scale bar represents the genetic distance.

**FIG 3:**
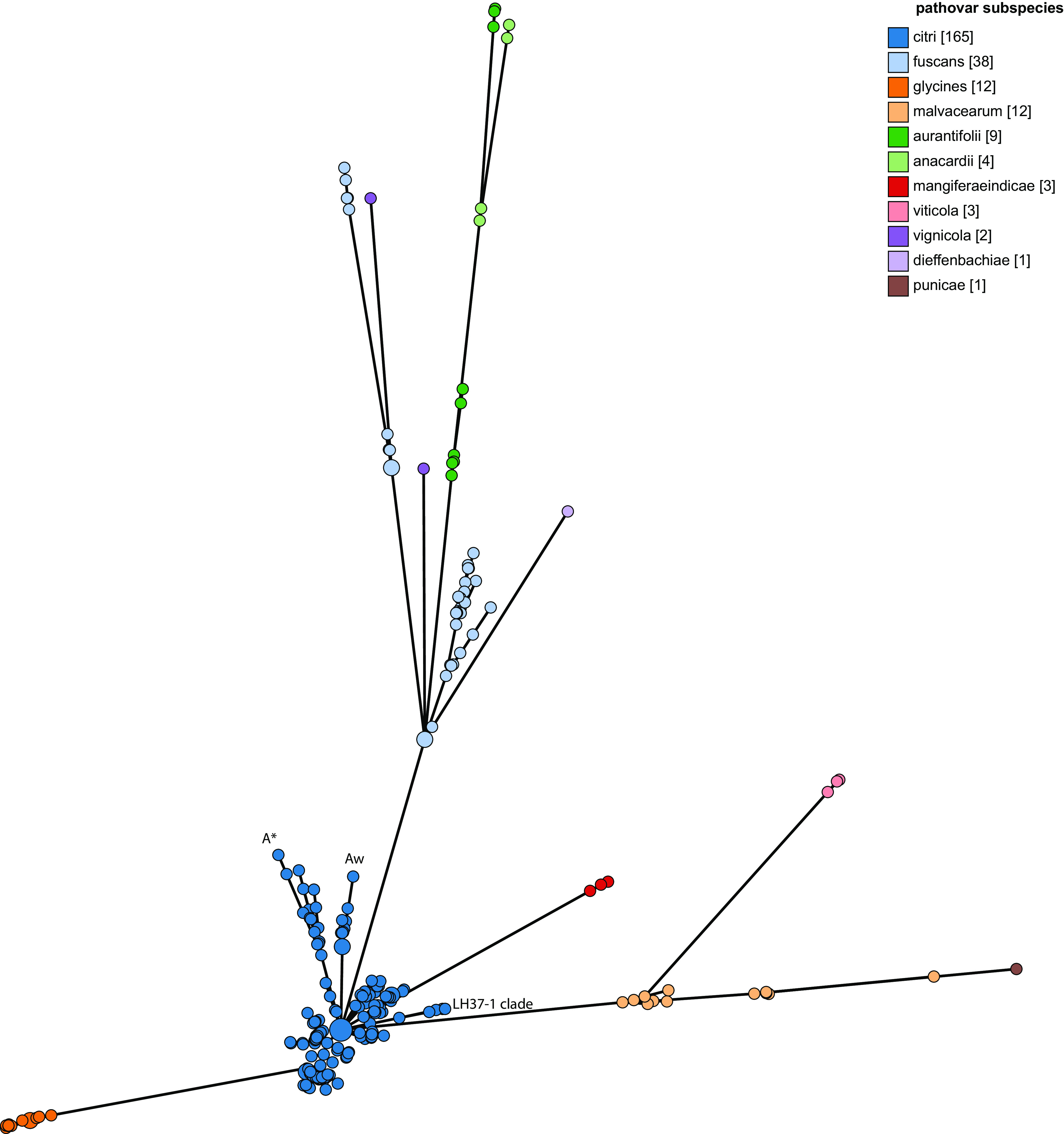
Minimum-spanning tree based on 250 core-genome allelic profiles of Xanthomonas citri. Isolates/groups are colored according to pathovar/subspecies. Pathotype A*, pathotype A^w^, and the divergent pathotype A clade containing five genomes, including that of isolate LH37-1, are shown. The phylogeny was generated using the GrapeTree (23) plug-in on the PubMLST website (https://pubmlst.org/).

A neighbor-joining tree of concatenated MLST allelic sequences of the 250 *X. citri* isolates is shown in Fig. 2. This phylogeny was generated using the Interactive Tree of Life (iTOL) plug-in on the PubMLST website. It clearly distinguishes individual *X. citri* pathovars (colored), with the genomes of isolates belonging to the same pathovar being grouped, although some subgroupings are evident. This is most marked for *X. citri* pv. *fuscans* isolate genomes, which are represented by three clades that include isolates from previous studies by Alavi et al. (12) and Aritua et al. (13). These correspond to isolates from three lineages originally named *X. citri* pv. *phaseoli* and *X. citri* pv. *phaseoli* GL 1 and *X. citri* pv. *phaseoli* GL *fuscans* GL2 and GL3. Genomes from isolates of *X. citri* pv. *aurantifolii* resolve as two main clades, with the smaller group showing more genetic similarity to members of the *X. citri* pv. *anacardii* group than to other members of *X. citri* pv. *aurantifolii*. Overall, this phylogeny displays a high degree of congruence with a neighbor-joining phylogeny based on core-genome SNPs, with the same groupings of genomes and only superficial differences in the tree structure (Fig. S1). A minimum-spanning tree generated using GrapeTree based on cgMLST allele data shows groupings identical to those made using both methods (Fig. 3).

The times taken to generate phylogenies based on core-genome SNPs using kSNP3 and MEGA 11 (36 h), concatenated allelic sequences using iTOL (15 min), and MLST allele data using GrapeTree (4 min) varied considerably. All tests were run on a 2020 3.6-GHz 10-Core Intel Core i9 iMac with 16 GB RAM.

### *X. citri* subsp. *citri* pathotype phylogenies.

**FIG 4:**
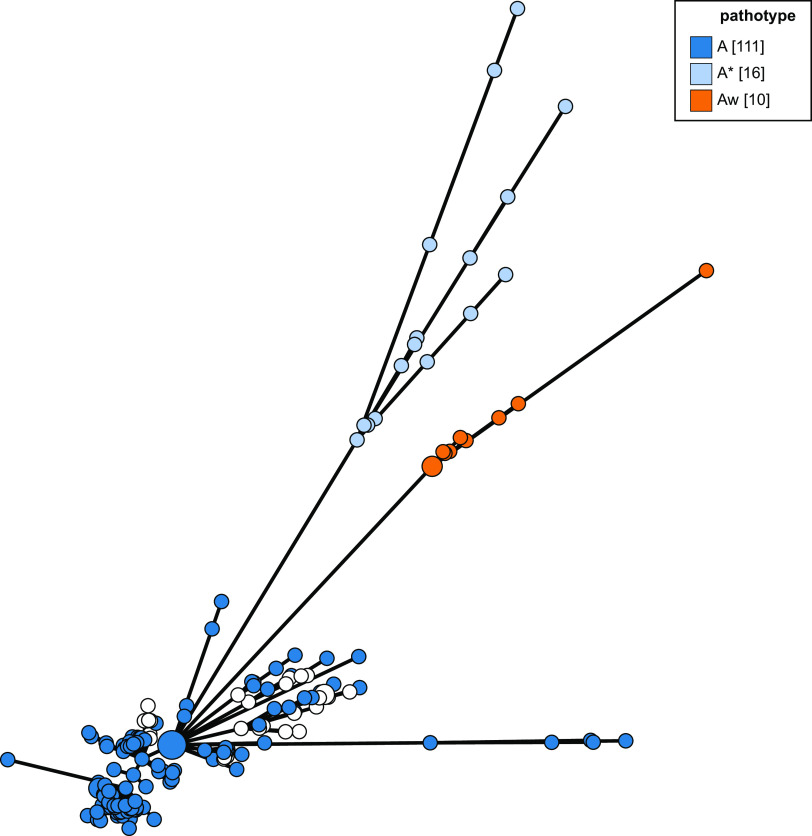
Minimum-spanning tree based on 127 Xanthomonas citri pv. *citri* core-genome allelic profiles, colored according to pathotype. The phylogeny was generated using the GrapeTree (23) plug-in on the PubMLST website (https://pubmlst.org/).

The genomes of pathotype A strains of *X. citri* pv. *citri* represented the largest group in this study, and these isolate genomes resolve as a discrete group in an NJ phylogeny based on concatenated cgMLST allele data (Fig. 2 and 3), with the exception of five isolates whose genomes were listed in the nucleotide submission information as being of pathotype A. These five isolates correspond to a separate lineage of pathotype A isolates examined in a previous study by Gordon et al. (4), which were isolated from grapefruit, key lime, and citrus species. Pathotype A* and A^w^ isolate genomes also form distinct clades using all three tree-building methods (Fig. 2). This can be seen in Fig. 2 and 3 but is clearer in Fig. 4 and Fig. S1, which include only *X. citri* subsp. *citri* isolate genomes.

## DISCUSSION

We have developed a cgMLST scheme for the study of Xanthomonas citri. Through its implementation on the PubMLST website, this scheme can be used to rapidly and robustly infer the pathovar (or subspecies) and, in the case of *X. citri* subsp. *citri*, pathotype designation(s) based on genetic similarity to uploaded and curated genomic sequence data with their associated metadata. The database currently contains 250 isolate genomes and their associated metadata, including 100 novel *X. citri* subsp. *citri* isolates sequenced in this study.

A neighbor-joining phylogeny based on concatenated allele sequences generated using the iTOL plug-in on the PubMLST website had a structure very similar to that of a core-genome SNP-based NJ tree but was generated within several minutes, compared to the >36 h required to generate an SNP-based phylogeny that depended on multiple-whole-genome alignment. This is important because as the number of sequenced *X. citri* genomes deposited in public databases increases, the computational resources required to generate SNP phylogenies *de novo* will become greater.

We used GrapeTree, implemented at PubMLST, to generate and display minimum-spanning trees of study data, and this plug-in can quickly and clearly display phylogenies colored according to metadata such as country of origin, date, host species, pathovar, and *X. citri* subsp. *citri* pathotype. The phylogenies generated using each of the methods used here, SNP-based NJ and iTOL and GrapeTree phylogenies based on concatenated cgMLST locus sequences, were largely congruent, with very similar groupings (see Fig. S1 in the supplemental material). However, GrapeTree, with its ability to easily and quickly display very large genomic data sets such as those present in EnteroBase, is eminently scalable as data sets grow, unlike core-SNP-based methods, which are more computationally intensive and time-consuming.

Neighbor-joining phylogenies based on concatenated rRNA gene sequences were generated in this study from genomes uploaded to the PubMLST website. This tree delineated the 21 different *Xanthomonas* species and 4 other more distantly related ones, including E. coli and P. aeruginosa. The rRNA gene is automatically applied to PubMLST genome data and serves as a further check of species designations for uploaded genome data.

The cgMLST scheme implemented on the PubMLST website for *X. citri* will, we hope, be an increasingly useful tool for the study of the epidemiology and evolution of the major cause of citrus canker, *X. citri* subsp. *citri*, but should also be of benefit for the study of other plant-pathogenic *X. citri* subspecies and pathovars included in this study as well as those not yet included in the database.

## MATERIALS AND METHODS

### Bacterial isolates.

A total of 101 *X. citri* subsp. *citri* isolates were obtained from Fundecitrus, Araraquara, São Paulo, Brazil, an association maintained by citrus growers and juice manufacturers from the State of São Paulo to conduct research, education, and implementation of citrus crop protection. Isolate 306, corresponding to the previously sequenced genome of strain 306 (14), was resequenced as part of this study, resulting in the sequencing of 100 novel isolates. These were sampled from citrus plants from 15 different countries and included 75 isolates from Brazil; 4 from South Korea; 3 each from Argentina and the United States; 2 each from China, New Zealand, and Paraguay; and 1 each from Australia, Fiji, France, India, Iran, Mauritius, Taiwan, Thailand, and Uruguay. One isolate’s country of origin is unknown. Details of the isolates are shown in Table 1. These isolates were all pathotype A isolates from sweet orange, with the exception of two pathotype A* isolates from key lime. Study bacteria were isolated between 1979 and 2015. Data on the year of isolation were not available for 31 of the 100 isolates.

### Genomic DNA sequencing.

Genomic sequencing was performed by MicrobesNG (University of Birmingham) from pure culture material stabilized in DNA/RNA Shield buffer (Zymo Research, CA, USA). Genomic DNA libraries were prepared using Nextera XT library prep kits (Illumina, San Diego, CA, USA). Libraries were sequenced using Illumina sequencers (HiSeq), using a 250-bp paired-end protocol. Reads were adapter trimmed using Trimmomatic 0.30 with a sliding window quality cutoff of *Q*_15_ (15) and scanned using Kraken (11) to confirm species identity. *De novo* assembly was performed on samples using SPAdes version 3.7 (16).

A further 150 *X. citri* genome sequences, downloaded from the European Nucleotide Archive (ENA), were included for analysis, including 65 *X. citri* subsp. *citri* isolates (comprising 12 pathotype A, 14 pathotype A*, and 10 pathotype A^w^ isolates according to their cited literature sources), 9 *X. citri* pv. *aurantifolii* isolates, 37 *X. citri* pv. *fuscans* isolates, 12 *X. citri* pv. *glycines* isolates, 12 *X. citri* subsp. *malvacearum* isolates, 3 *X. citri* pv. *mangiferaeindicae* isolates, 3 *X. citri* pv. *viticola* isolates, 2 *X. citri* pv. *vignicola* isolates, and 1 isolate each of *X. citri* pv. *dieffenbachiae* and pv. *punicae*. Details of all *X. citri* genomes included in this study are shown in Table 1. In addition, 24 genomes representing single examples of 20 different *Xanthomonas* spp. and single examples of Stenotrophomonas maltophilia, Escherichia coli, Xylella fastidiosa, and Pseudomonas aeruginosa were downloaded from GenBank. Details of these isolates are shown in Table S1 in the supplemental material.

### Core-genome MLST.

Complete coding sequences were identified in the finished genome assembly of strain 306 (14) using Prokka (17) with default settings. These were used in Roary (18) to identify 1,618 genes found in all 250 genomes. A BIGSdb database for *X. citri* was set up on the PubMLST website (19), with loci being defined for each of the identified core genes and named using an XCIT prefix and a five-digit identifier, ranging from XCIT00001 to XCIT01618. The database was seeded with the coding sequence found in strain 306 for each of these loci defined as allele 1. Allelic variants found in the 100-isolate locally sequenced data set were then identified using the BIGSdb allele caller, with thresholds of 98% identity over 98% of the alignment length compared to reference alleles. A further round of allele calling using the same parameters and all previously identified alleles as references was performed, followed by manual scanning to identify more variable alleles containing small indels. The database was then expanded to include all 250 isolates and alleles identified as described above. Start codon positions were adjusted in nine loci as the codon identified in the reference genome was not found consistently across the data set, whereas an alternate consensus start codon was identified nearby. Core-genome sequence types (cgSTs) were defined automatically by BIGSdb for profiles with fewer than 50 missing loci. Single-linkage cluster schemes were set up within the database to identify related isolates using a range of locus mismatch thresholds (200, 100, 50, 25, 10, and 5 locus mismatches).

Ribosomal MLST (rMLST) (20), implemented on the PubMLST website, confirmed the species identity of all 274 study isolates. It was also used to generate concatenated rRNA gene sequences for phylogenetic analysis. As rMLST examines allelic variation at 53 universal rRNA genes, it is ideally suited for the rapid phenotypic analysis of genomes of different species.

### Phylogenetic trees.

In common with previous studies of *X. citri* evolution and epidemiology, we generated phylogenies based on core-genome SNPs using a reference genome. We used the finished genome of *X. citri* pv. *citri* strain 306 (14) as a reference and kSNP3 v3.12 (21) to generate fasta nucleotide files of SNPs, which were used in MEGA 11 (22) to generate NJ trees. Genomic DNA sequence data and their associated metadata can be analyzed using a variety of methods implemented on the PubMLST website. Here, we generated minimum-spanning trees from allelic profiles using GrapeTree (23). Neighbor-joining trees based on concatenated nucleotides of cgMLST loci were generated using Interactive Tree of Life (iTOL) (24). Both the GrapeTree and iTOL plug-ins are implemented on the PubMLST website (https://pubmlst.org/). A neighbor-joining tree was constructed for the 250 *X. citri* study isolates and 24 other species listed in Table S1. This was generated from the 53 concatenated rRNA gene sequences used in the rMLST scheme using the iTOL plug-in as described above.
