# Supplementary material for: Core-Genome Multilocus Sequence Typing for Epidemiological and Evolutionary Analyses of Phytopathogenic Xanthomonas citri
Source: Appl Environ Microbiol. 2023 Apr 17;89(5):e02101-22. doi: 10.1128/aem.02101-22 (PMC10231234; doi:10.1128/aem.02101-22)
Supplement: Supplemental file 1 — Supplemental material. Download aem.02101-22-s0001.pdf, PDF file, 0.2 MB [file aem.02101-22-s0001.pdf]

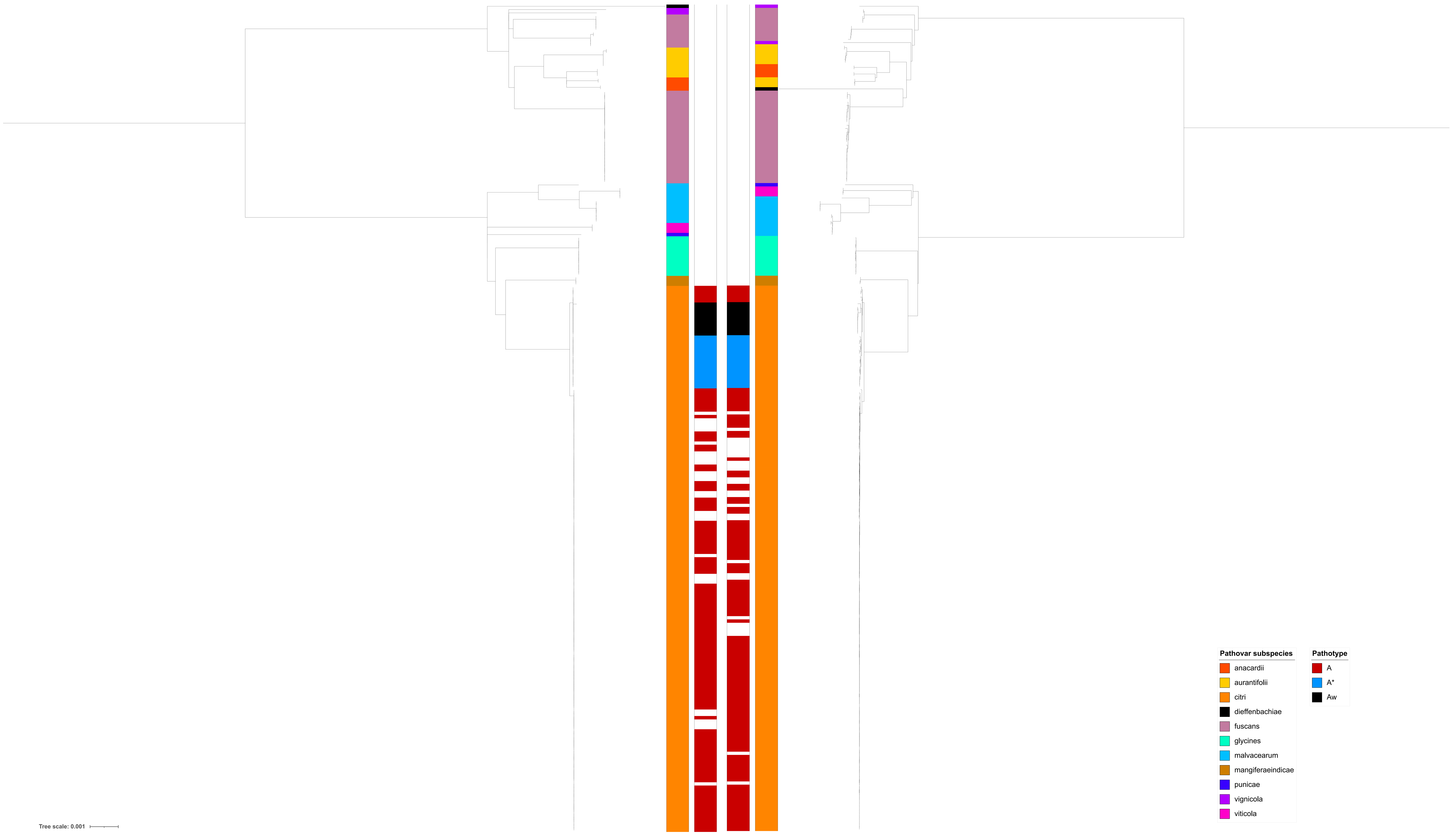

Supplementary Figure 1. Comparison between neighbour-joining trees of 250 Xanthomonas citri isolate genomes generated (left) using concatenated MLST allele sequences and (right) core-genome SNPs coloured according to subspecies and Xanthomonas citri pv. pathotype. Scale bar represents genetic distance.

Suppl. Table 1. Details of additional genome sequences used in ribosomal MLST analysis.

Species	Genbank Accession	Strain
Escherichia coli	GCF_000005845.2	K-12
Pseudomonas aeruginosa	GCF_000006765.1	PAO1
Xylella fastidiosa	GCF_000007245.1	Temecula1
Xanthomonas albilineans	GCF_000087965.2	GPE PC73
Xanthomonas vasicola	GCF_000277895	NCPPB 4379
Xanthomonas floridensis	GCF_001642575.1	WHRI 8848
Xanthomonas nasturtii	GCF_001660815.1	WHRI 8853
Xanthomonas vesicatoria	GCF_001908725.1	LMG911
Xanthomonas hortorum	GCF_002285515.1	B07-007
Xanthomonas dyei	GCF_002939865.1	CFBP7245
Xanthomonas melonis	GCF_002940015.1	CFBP4644
Xanthomonas sacchari	GCF_002940085.1	CFBP4641
Xanthomonas oryzae	GCF_008370835.2	GX01
Xanthomonas sontii	GCF_009649705.1	ASD011
Xanthomonas campestris	GCF_013388375.1	MAFF106181
Xanthomonas translucens	GCF_017301775.1	XtLr8
Xanthomonas euvesicatoria	GCF_017724035.1	CFBP3836
Xanthomonas perforans	GCF_020879715.1	DC05T6
Xanthomonas prunicola	GCF_025266575.1	CIX249
Xanthomonas fragariae	GCF_900183975.1	PD885
Stenotrophomonas maltophilia	GCF_900475405.1	NCTC10258
Xanthomonas euroxanthea	GCF_903989455.1	CPBF 424
Xanthomonas hydrangeae	GCF_905142465.1	GBBC 2199
Xanthomonas arboricola	GCF_905367715.1	CPBF 1494
